# A novel synthetic derivative of quercetin, 8-trifluoromethyl-3,5,7,3′,4′-*O*-pentamethyl-quercetin, inhibits bladder cancer growth by targeting the AMPK/mTOR signaling pathway

**DOI:** 10.18632/oncotarget.17799

**Published:** 2017-05-11

**Authors:** Ting Tao, Caimei He, Jun Deng, Yanjun Huang, Qiongli Su, Mei Peng, Meiling Yi, Kwame Oteng Darko, Hui Zou, Xiaoping Yang

**Affiliations:** ^1^ Key Laboratory of Small Targeted Molecules’ Discovery, Hunan Normal University, Changsha, Hunan, 410013 China; ^2^ Department of Pharmacy, School of Medicine, Hunan Normal University, Changsha, Hunan, 410013 China; ^3^ Key Laboratory of Animal Nutrition and Human Health, Hunan Normal University, Changsha, Hunan, 410013 China; ^4^ Department of Pharmacy, Xiangya Hospital, Central South University, Changsha, Hunan, 410008 China

**Keywords:** 8-Trifluoromethyl-3,5,7,3′,4′-O-pentamethyl-quercetin, quercetin, derivative of quercetin, AMPK, mTOR

## Abstract

Quercetin is a naturally existing compound and shows attractive anticancer properties for a variety of solid tumors including glioma, bladder cancer, hepatocellular carcinoma, breast cancer, hematological malignancies and prostate carcinoma. However, these anticancer properties have not been clinically approved due to unclear mechanistic information and its low bioactivity. In our previous study, we elucidated that quercetin activates AMPK pathway which is the major mechanism for its unique anticancer effect in bladder cancer. In the present study, we are trying to enhance its bioactivity by chemical modification using fluorination approach to prepare novel chemical entities, based on the principle of intermediate derivative method (IDM). The compound we obtained is named 8-trifluoromethyl-3,5,7,3′,4′-O-pentamethyl- quercetin (TFQ), characterized by NMR spectra and mass spectrum (MS). The results from MTT and cologenic assay in two human and one murine bladder cancer cell lines showed that TFQ exhibits more potent inhibition on the three bladder cancer cell lines than quercetin (Que) although this enhanced effects is not very dramatic. Furthermore, we found that the survival of normal bladder cells PEBC was not significantly suppressed by TFQ compared with Que. Western blot analysis showed that TFQ possess more potent AMPK activation than Que. The downstream of AMPK was further examined by western blot. TFQ treatment is able to inactivate mTOR signaling pathway with the regulation of mTOR, 4EBP1 and P70S6K. These results demonstrated that the fluorinated quercetin derivative TFQ inhibits bladder cancer cell growth through the AMPK/mTOR pathway. Altogether, our findings suggest that TFQ could serve as a new potential therapeutic agent for bladder cancer more effective than Que.

## INTRODUCTION

Bladder cancer is the fifth most common malignancy in United States, with an estimated 76,960 new cases and 16,390 deaths in 2016 [1–2]. Even though transurethral resection has served as the standard treatment, recurrence and metastasis are often seen in clinic [3]. The commonest way to prevent recurrence and progression is supplemented with intravesical chemotherapy or immunosuppressive agents [4, 5]. Despite these brave efforts, outcomes have changed very little for the past three decades [6]. Metabolism of bladder cancer represents a key issue for cancer research. Several metabolic altered pathways are involved in bladder tumorigenesis [7].

Quercetin is a naturally existing flavonoid in several vegetables and fruits with attractive biological activities [8]. It has been demonstrated that this small molecule has extensive pharmacological effects, including antitumor, antioxidative and anti-inflammatory activities [9, 10]. The anticancer activity of quercetin has been shown in many kinds of cancers including breast cancer [11, 12], pancreatic cancer [13], medulloblastoma [14], myeloma [15], glioma [16] and bladder cancer [17, 18].

In United States, quercetin has been approved as a non-prescription drug widely used to treat prostate cancer. However, quercetin alone has poor bioavailability [19]. Thus, people have contributed fairly amount of efforts to modify the chemical structure of quercetin for the purpose of obtaining more active compounds via etherification [20], esterification [21], Mannich reaction [22] and metal complexes of quercetin [23, 24]. In a previous study, we reported the synthesis of brominated quercetin derivatives [25]. However, these compounds did not show any promising value for further investigation (data not shown). Based on the concept of intermediate derivative method (IDM), which supposedly finds novel bioactive compound efficiently [26], we are trying to determine whether there is any benefit of fluorination on quercetin due to that fluorinated compounds have become very successful chemical entities for clinical application [27–29]. There are no reports regarding the fluorination of quercetin. In this study, we are trying to enhance its bioactivity by chemical modification using fluorination approach to obtain novel chemical entities. The compound we synthesized is named 8-trifluoromethyl-3,5,7,3′,4′-O-pentamethyl-quercetin (TFQ) and was evaluated its antitumor activities and molecular mechanisms.

In a previous study, we elucidated that quercetin activates AMPK pathway which may be a major mechanism for its unique anticancer effects [30]. AMPK is a metabolic sensor in the cell activated by a high AMP: ATP ratio and low nutrient availability and signals to shut off anabolic processes such as protein synthesis in favor of catabolic processes such as fatty acid oxidation [31]. AMPK plays an important role in regulating cellular metabolism, preserving cellular energy homeostasis, and is also involved in many other cellular processes, including cell apoptosis [32, 33]. Recently, emerging evidence has implicated the potential roles of AMPK signaling in tumor initiation and progression [34]. Furthermore, AMPK has been considered as a potential therapeutic target for the treatment of cancer [35]. Activation of AMPK induces cell cycle arrest and inhibits cell survival in malignant cells through multiple mechanisms including up-regulation of the p53-p21 axis [36], and down-regulation of the mammalian target of rapamycin (mTOR) pathway [37, 38].

In our quest to develop novel quercetin derivatives with increased potency for AMPK activation and mTOR inhibition, we found that compared with quercetin, TFQ activated AMPK with significantly greater potencies. Therefore, it would be an attractive approach to develop novel AMPK activators as anti-cancer agents from structure-modified natural compound, which possess higher efficacy, lower toxicity.

## RESULTS

### Chemistry

**Scheme 1:**
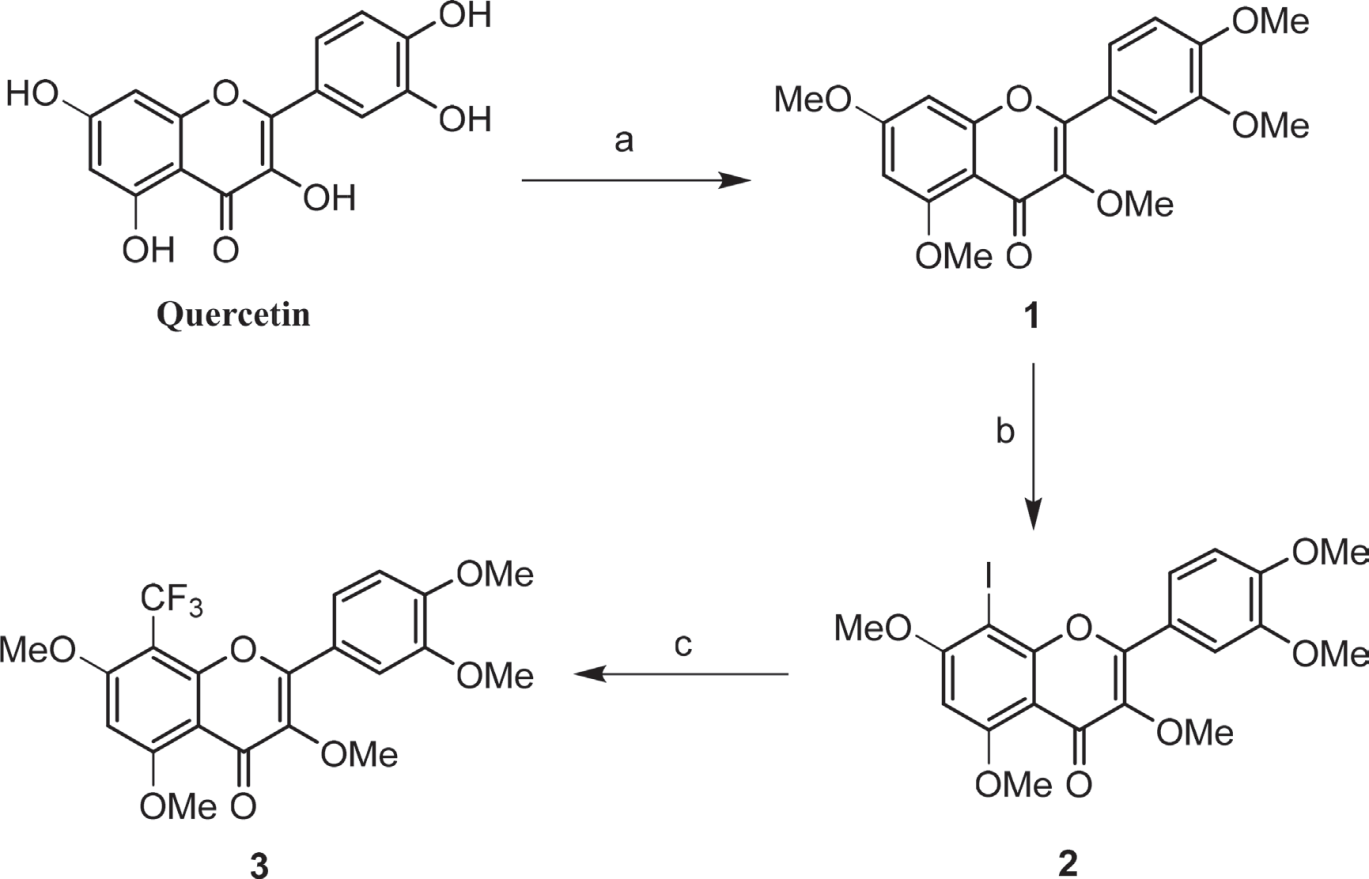
Synthetic route of TFQ (3): (**a**) K_2_CO_3_, CH_3_COCH_3_, CH_3_I, reflux; (**b**) ICl, AcOH/DMSO, 55°C; (**c**) FSO_2_CF_2_CO_2_Me, CuI, HMPA/DMF, 75°C.

**Figure 1:**
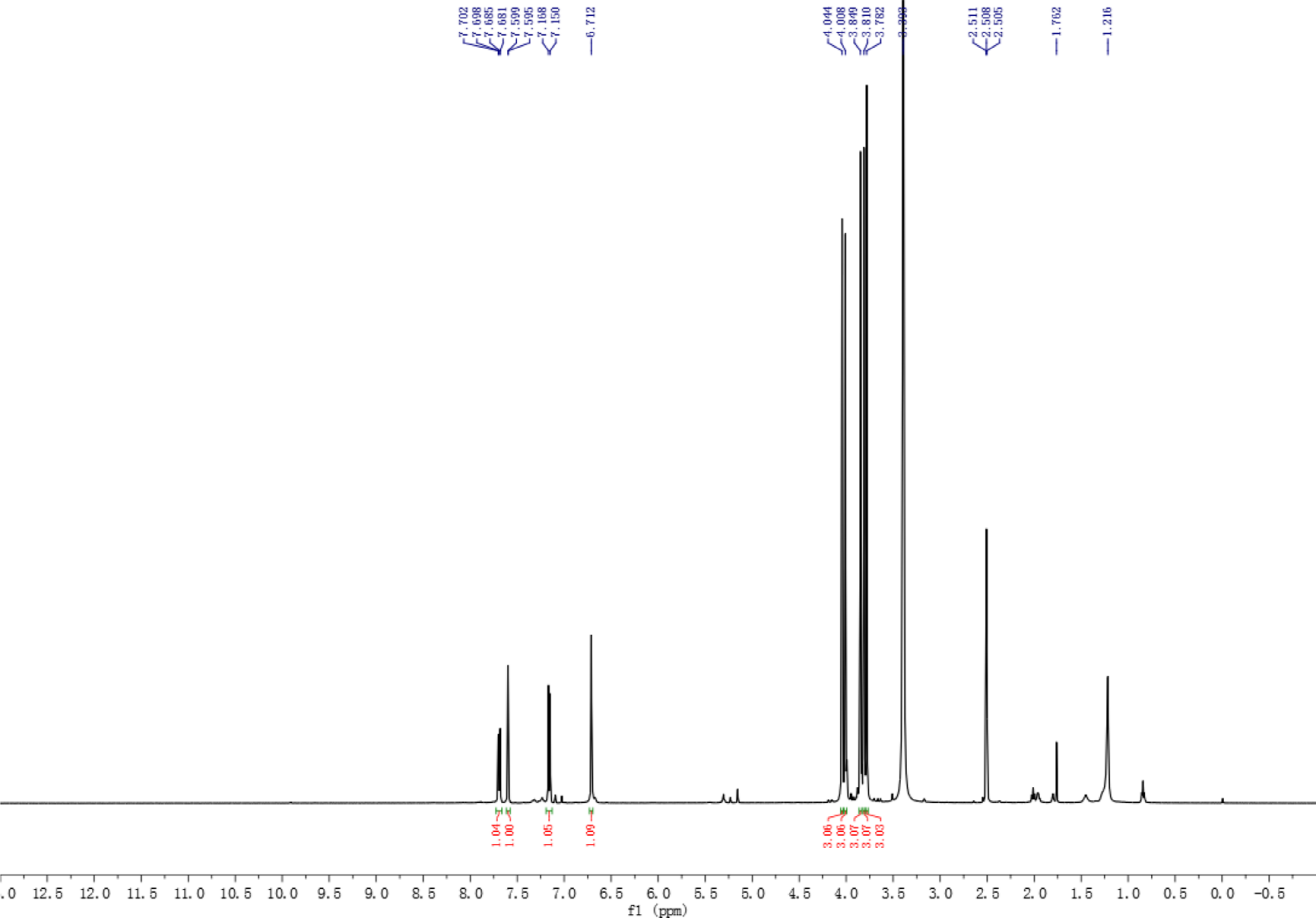
^1^H NMR spectrum of compound 3

**Figure 2:**
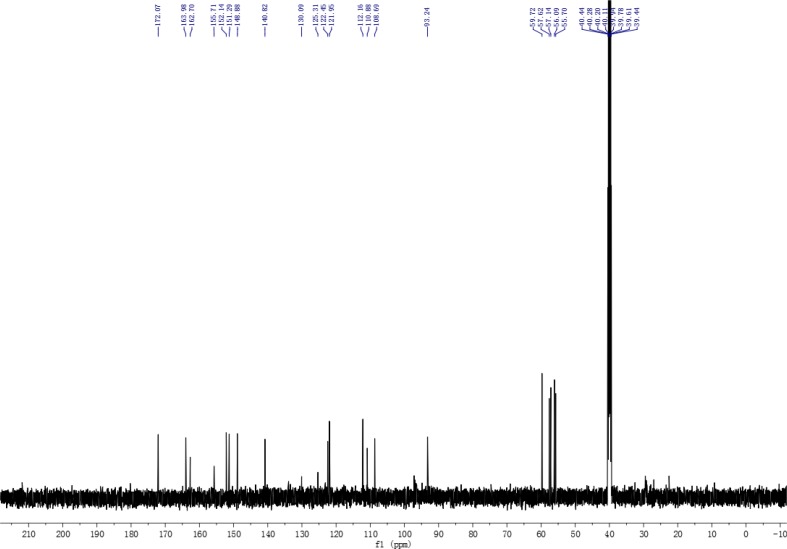
^13^C NMR spectrum of compound 3

The TFQ (3), a derivative of quercetin containing a trifluoromethyl group, was prepared by following the expected reaction route in Scheme 1. Thus, reaction of the commercially available quercetin with iodomethane afforded the known full methylated product (1) [39]. The product were iodinated with ICl in dry DMSO to yield compound 2 [40]. The product 2 was then reacted with the trifluoromethyl reagent (methyl fluorosulfonyldifluoroacetate) under nitrogen protection in the presence of CuI/DMF/HMPA afforded the desired compound 3 after purification by prep-HPLC [41]. Compound 3 was elucidated as 8-trifluoromethyl-3,5,7,3′,4′-O-pentamethyl-quercetin by NMR and HRESIMS techniques. The NMR spectra of compound 3 were showed in Figures 1 and 2, and the NMR data of compounds 1, 2 were listed in supplemental materials.

### TFQ inhibits bladder cancer cell survival

**Figure 3:**
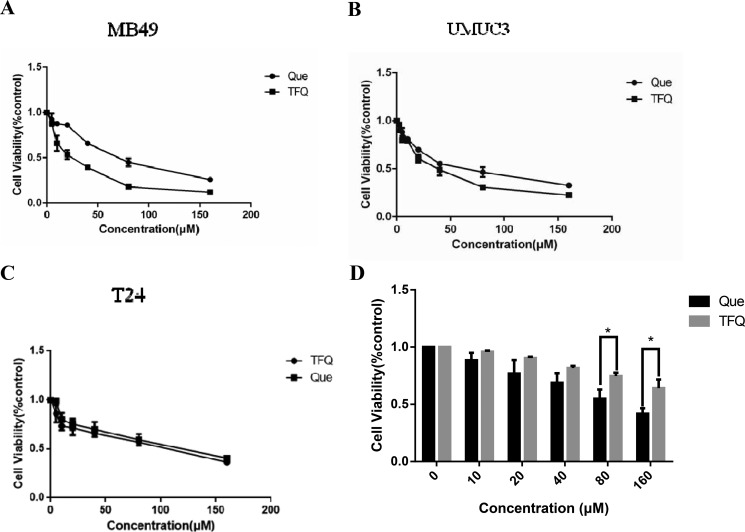
Treatment with Queor TFQ on cell survival of 3 bladder cancer cell lines (**A**) TFQ or Que inhibited MB49 survival synergistically. Cell viability was assessed after a 48 hour. Que or TFQ treatment at concentrations ranging from 0 to 160 μM using a tetrazolium-based assay. (**B**–**C**) TFQ or Que inhibited T24 and UMUC3 survival synergistically. Cell viability was assessed with 48 hour Que or TFQ at 0, 10, 20, 40, 80, 160 μM treatment in T24 and UMUC3, respectively. (**D**) TFQ or Que inhibited normal cell of PEBC survival synergistically. Results are presented as the median of 5 independent experiments.

Three bladder cell lines (MB49, UMUC3, T24) were exposed to 0∼160 μM TFQ or quercetin (Que) to evaluate their inhibition on the survival. The results demonstrated that TFQ exerted more potent cytotoxic effects against MB49 (IC_50_ 44.69 μM), UMUC3 (IC_50_ 72.65 μM) and T24 (IC_50_ 100.46 μM) cell lines compared to Que (IC_50_ 72.45, 94.69 and 118.91μM, respectively) (Table 1 and Figure 3A–3C) although the differences are mild. The survival of normal cells PEBC was not significantly inhibited when treated with TFQ for 48 h compared with Que, suggesting that TFQ had much less cytotoxic effect on normal cells than Que, as shown in Figure 3D.

**Table 1:** Inhibitory concentration 50% (IC_50_) of Que and TFQ

IC_50_ value	IC_50_ value	
	TFQ	Que
MB49	44.69	72.45
UMUC3	72.65	94.69
T24	100.46	118.91
PEBC	168.26	105.72

### TFQ suppresses colony formation

**Figure 4:**
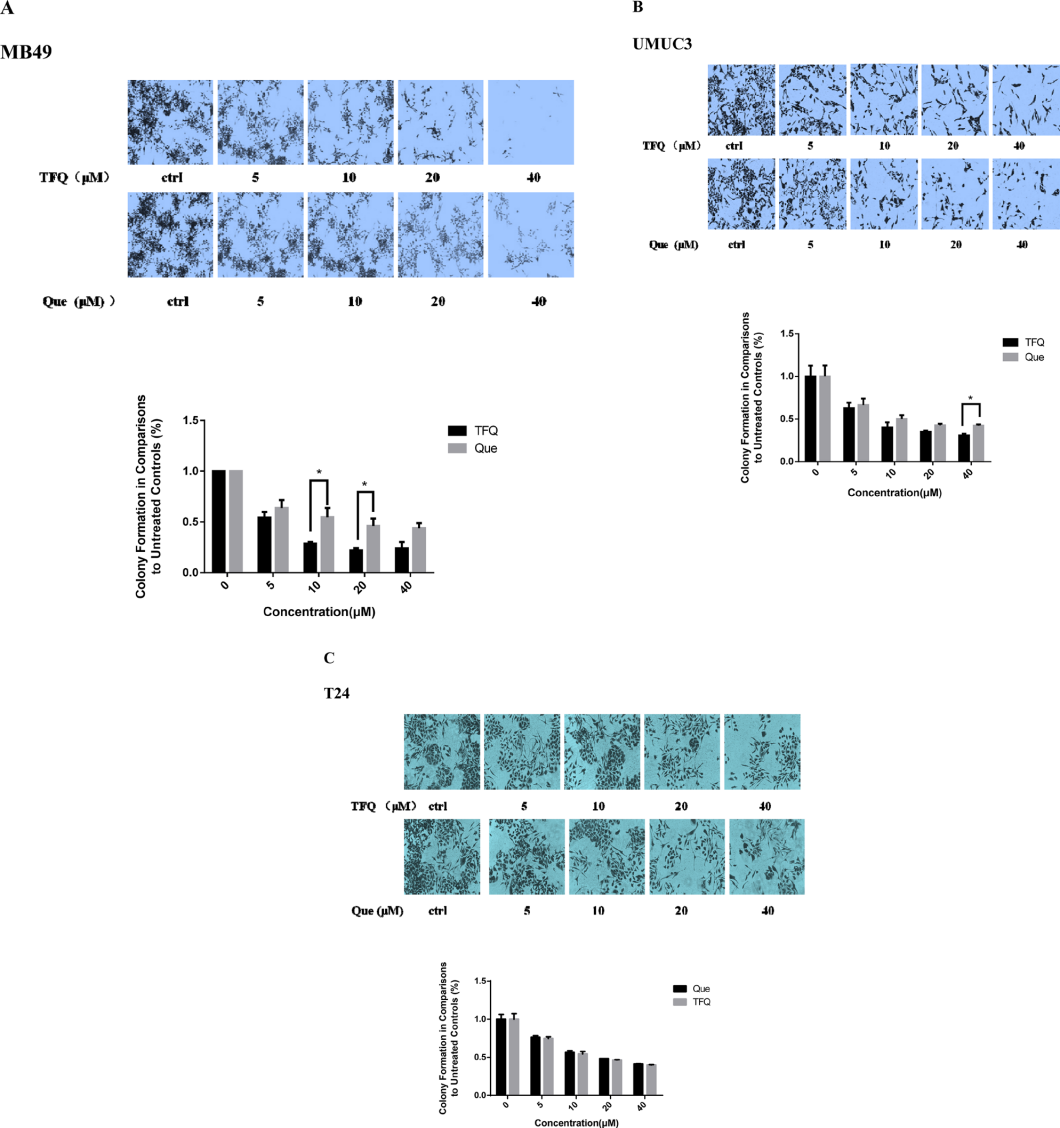
Evaluation of colony suppression of TFQ or Que on 3 bladder cancer cell lines (**A**–**C)** clonogenic assay was assessed after 7 day TFQ or Que treatment and wells were stained with crystal violet at the end of the experiment. A. Clonogenic assay in MB49 was conducted with the assessed after 7 day treatment at various concentrations (0–40 μM) TFQ or Que. Above: The full view of wells were taken through stereomicroscope and images were taken through an inverted microscope with × 10 magnification. Below: the quantification of colony was determined by microplate area scan at OD 550 nm, Results are presented as the median of 5 independent experiments (Comparison was draw by the *t*-test (two-tailed). Data represent means ± SD; TFQ vs Que). (B–C) clonogenic assay was conducted with the treatment at various concentrations (0–40 μM) TFQ or Que in T24 and UMUC3, respectively. the full view of wells and their quantification were obtained through the same method as described in MB49. Results are presented as the median of 5 independent experiments (*P < 0.05, TFQ vs Que).

Colony formation was examined in the presence of TFQ or Que with the range of 0–40 μM. As shown in Figure 4A–4C, colony numbers were significantly decreased in the three bladder cancer cells. The results confirmed the TFQ exerted a better suppression effect on bladder cancer cell survival or motility than Que in MB49 and UMUC3 cell lines but there was no significant difference in T24 cell line.

### Scratch wound healing assay

**Figure 5:**
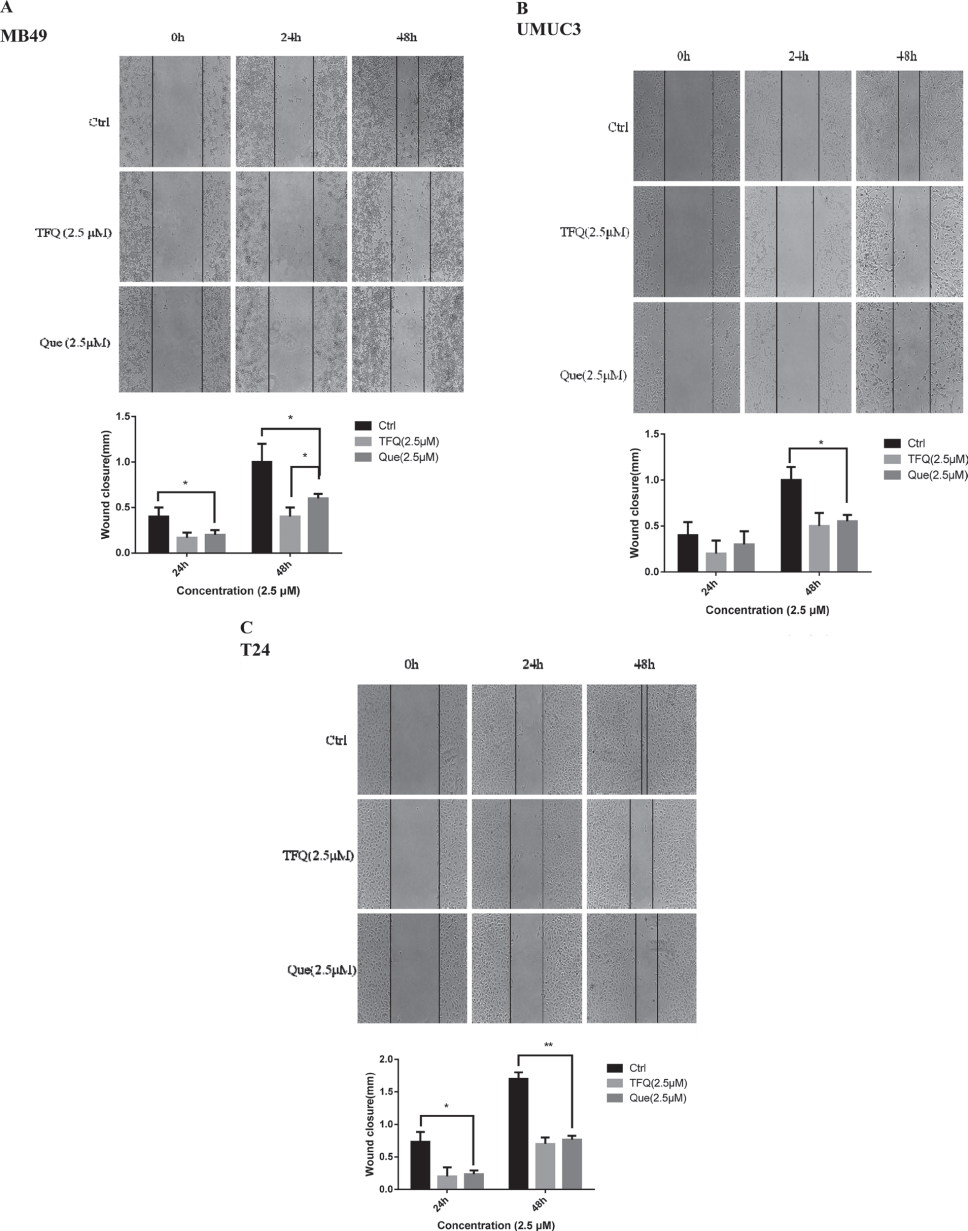
TFQ or Que treatment showed impaired migration in wound healing assays (**A**–**C**) Images showed the gap of the scratched region of different cells with various treatments; A. Wound healing assays in MB49 conducted with the treatment of 5μM TFQ or Que. Above:The images were taken through an inverted microscope with × 10 magnification. Below: The mean area was calculated using Image J software. Results are presented as the median of 5 independent experiments (*P < 0.05 vs control and TFQ vs Que). (B–C) Wound healing assays was conducted with the treatment at 2.5 μM TFQ or Que in T24 and UMUC3, respectively. The images and their mean area were obtained through the same method as described in MB49.

Cell scratch assay was used to analyze whether TFQ or Que affected the migration of bladder cancer cells. As shown in Figure 5, at a low concentration of 2.5 μM, cell free area of the TFQ-treated group was significantly wider than both the control and Que-treated groups in all three bladder cancer cell lines at 24 and 48 hours respectively. There was a dramatic difference in control and Que-treated groups of MB49 but there was no significant difference in T24, UMUC3. Similar pattern was observed in high concentration treated groups (In Supplementary Materials).

### Effects on apoptosis with treatment of TFQ

**Figure 6:**
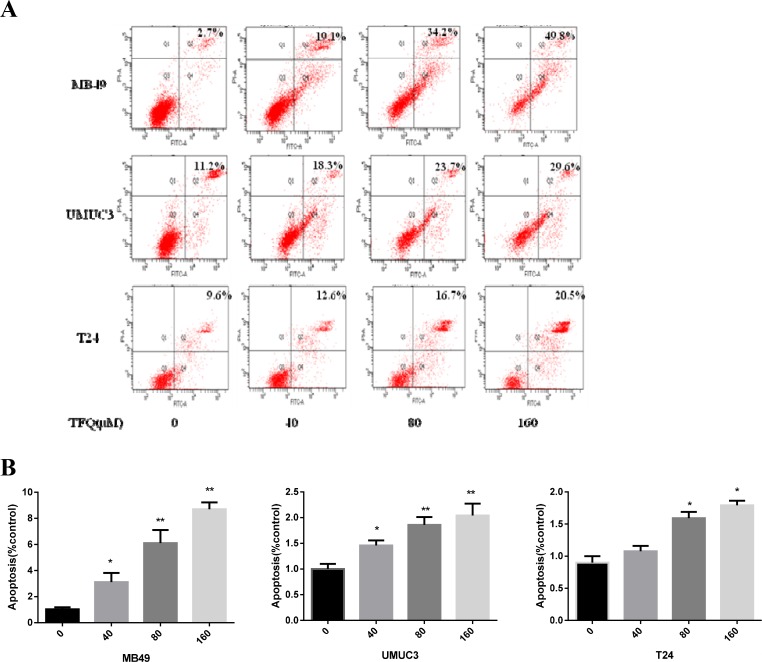
TFQ induces apoptosis in bladder cancer cell lines in a dose dependent manner (**A)** Representative flow cytometry scatter plots of propidium iodide (PI) (Y axis) vs Annexin V-fluorescein isothiocyanate (FITC) (X axis). (**B**) Bar chart shows quantitative data of average of 3 independent flow cytometry experiments *P < 0.05, **P < 0.01 compared with control.

Our previous studies have shown that quercetin can induce apoptosis [30]. We next examined whether TFQ could induce apoptosis as well. Two human bladder cancer cell lines and one murine bladder cancer cell line were treated with TFQ (0, 40 μM, 80 μM, 160 μM) for 24 h and apoptosis was measured by Annexin V/PI staining. In this experiment, we found that TFQ led to a dose-dependent cell apoptosis. As shown in Figure 6, cellular apoptosis was considerably increased in the TFQ-treated groups compared to the control group in a dose-dependent manner. After treatment with 160 μM TFQ, the apoptotic populations increased as follows: from 7.6% to 49.8% in MB49 cell line, from 11.% to 29.6% in UMUC3 cell line, and from 9.6% To 20.5% in T24 cell line. These results demonstrated that TFQ is able to induce apoptosis in bladder cancer cells.

### TFQ activates AMPK

**Figure 7:**
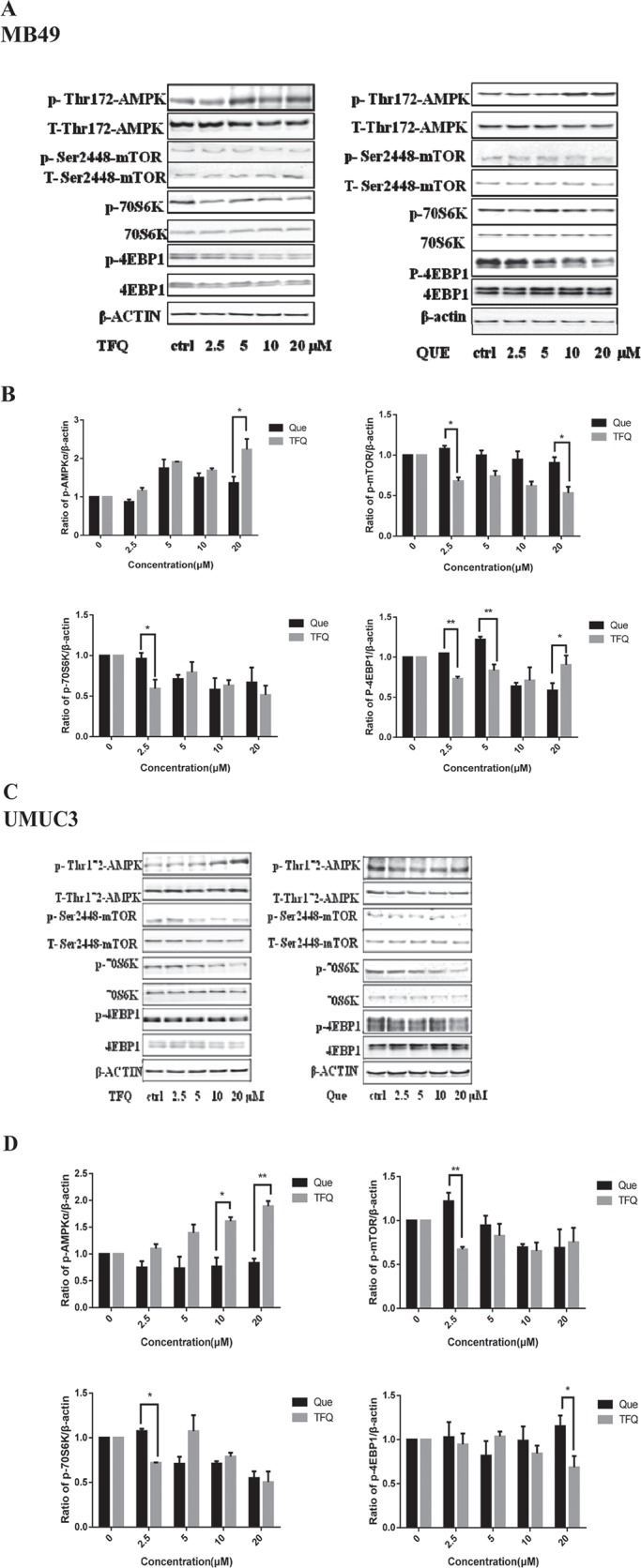

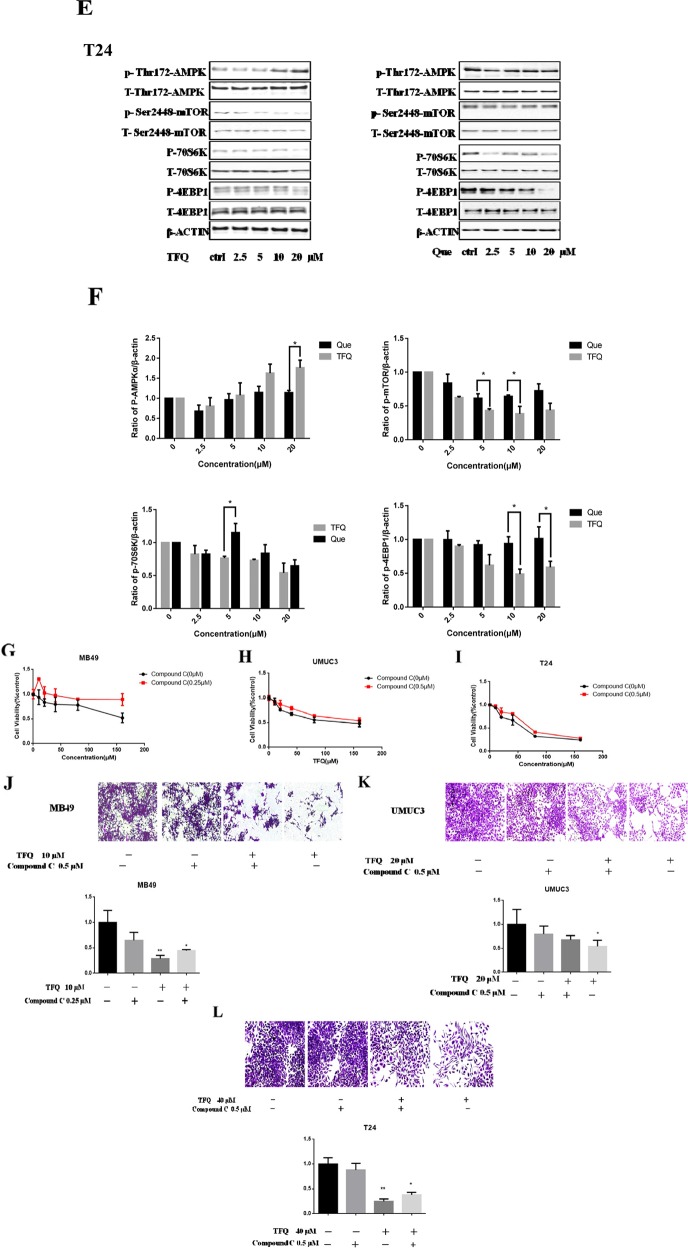
TFQ activates AMPK/mTOR intracellular signaling pathways at various concentrations (**A)** Shows western blot of p-AMPK, p-p70S6K, p-mTOR, p-4EBP1, t-AMPK, t-mTOR, t-p70S6K and t-4EBP1 with the treatment of TFQ or Que (0–20 μM) in MB49. β-actin was included as a loading control. (**B)** Shows the ratio of proteins of interests to β-actin calculated by the band density of western blots using Image J software of MB49 cell line. (*P < 0.05, **P < 0.01 TFQ vs Que). Data were presented as cell survival curves. TFQ vs Que (**C**) Shows western blot results of p-AMPK, p-mTOR, p-P70S6K, p-4EBP1, t-AMPK, t-mTOR t-P70S6K and t-4EBP1 with the treatment of TFQ or Que (0–20 μM) in UMUC3. (**D**) Shows the ratio of proteins of interests to β-actin calculated by the band density of western blots using Image J software of UMUC3 cell line. (*P < 0.05, **P < 0.01 TFQ vs Que). (**E**) Shows western blot results of p-AMPK, p-mTOR, p-P70S6K, p-4EBP1, t-AMPK, t-mTOR t-P70S6K and t-4EBP1 with the treatment of TFQ or Que (0–20 μM) in T24. (**F**) Shows the ratio of proteins of interests to β-actin calculated by the band density of western blots using Image J software of T24 cell line. (*P < 0.05, **P < 0.01 TFQ vs Que). (**G**–**I)** Cell viability was assessed after 48 hour TFQ alone or TFQ combined with 0.25 μm AMPK antagonist compound C treatment in MB49 and treatment at 20 μM TFQ alone or TFQ combined with 0.5 μm AMPK antagonist compound C in T24 and UMUC3, respectively. Results are presented as the median of 5 independent experiments. Compound C significantly reduced the decrease of cell survival and colony suppression caused by TFQ, (**J**–**L**) Clonogenic assay was assessed after 7 day TFQ treatment alone or combined with compound C and wells were stained with crystal violet at the end of the experiment. J. Clonogenic assay in MB49 was conducted with the treatment of 10 μM TFQ, 0.25 μM Compound C and their combination. (K–L) Clonogenic assay in MB49 was conducted with the treatment of 20 μM TFQ, 0.5 μM Compound C and their combination in UMUC3 and T24, respectively. Above: The full view of wells were taken through stereomicroscope and images were taken through an inverted microscope with × 10 magnification. Below: the quantification of colony was determined by microplate area scan at OD 550 nm, Results are presented as the median of 5 independent experiments (Comparison was draw by the *t*-test (two-tailed). Data represent means ± SD; *P < 0.05,**P < 0.01 vs control).

AMPK, a central energy sensor, is known as a crucial sensor in controlling metabolism and cancer cell growth. Previous study has elucidated that quercetin activates AMPK pathway which may be a major mechanism for its unique anticancer effect [30]. We compared the effects of TFQ and Que in this regard. Three bladder cancer cell lines (MB49, UMUC3 and T24) were treated with TFQ or Que with same dose in the range of 0-20 μM for 2 h, and cell lysates were analyzed for their levels of p-AMPK (T172), p-mTOR (S2448) and the mTOR downstream effectors p70S6K and p4EBP1 by Western blot analysis. The results revealed that TFQ activates AMPK with significantly greater potencies than Que as shown in Figure 7A–7F. This suggests that TFQ might enhance the pharmacological activities of Que with the activation of AMPK and influence on AMPK downstream. There was significant difference in the inhibitory effect of mTOR downstream effectors 4EBP1 and p70S6K after treated TFQ or Que 2 hours.

Next, we utilized AMPK specific inhibitor compound C to determine whether activating AMPK signaling pathway is essential for TFQ to exert anti-tumor effect. As shown in Figure 7G, the ability of TFQ to inhibit MB49 cancer cell survival was reduced after been treated with compound C, implying the inhibition of AMPK accelerates survival of cancer cells. Similar trends of human bladder cancer cells T24 and UMUC3 were observed as shown in Figure 7H–7I, consistent with colony formation results as shown in Figure 7K–7L.

## DISCUSSION

Bladder cancer remains one of the main malignancies that affect the genitourinary tract [42]. Nowadays, chemotherapy is the main treatment but recurrence and metastasis are often seen in the clinic which is becoming the most common problem. Therefore, fairly amount of research groups are focusing on studying natural products, produced by secondary metabolism of plants, in an attempt to find new and better treatments to tumors. Quercetin, a widely distributing member of natural flavonoids, is well known for its anticarcinogenic potential. It has been reported that that quercetin possesses strong anticancer activities against a variety of cancer cell lines including solid cancer [11–18]. Further study by chemical modifications to increase its efficacy could further its applicability and discover more effective candidate molecules. It is well known that the introduction of CF_3_ group into organic molecules often improves their physiological, physical chemical properties without the introduction of extra steric hindrance. Moreover, trifluoromethylation frequently improve the bioactivity. So far, we have studied the trifluoromethylation of quercetin for the first time.

The present study reported the synthesis of a novel trifluoromethylated derivative of quercetin, named TFQ. Previously, we reported that quercetin induced bladder cancer cells apoptosis by activation of AMPK signaling pathway [30]. Although the precise underlying mechanism(s) have not yet been fully elucidated, increasing evidence indicates that the P13K/Akt/mTOR pathway plays a crucial role in the survival and survival of cancer cells [43, 44]. Interestingly, compared with Que, TFQ activated AMPK and inactivated mTOR with significantly greater potencies in all of the tested bladder cancer cell lines. Accumulating evidence indicates that AMPK activation strongly suppresses cell survival, and further confirmed by AMPK inhibitor compound C. It has been proved that AMPK phosphorylation can induce TFQ-induced cell viability inhibition and inhibits bladder cancer progression. This is shown by colony formation assays, wound healing assays and apoptosis. Notably, TFQ displays low cytotoxicity to human bladder normal cells. In conclusion, we herein confirm that the fluorinated quercetin derivative, TFQ blocks bladder cancer cells growth and increases apoptotic progression more effective than Que via activating AMPK and blocking the mTOR pathways. TFQ may be a useful small molecule candidate for the treatment of bladder cancer via AMPK/mTOR signaling pathway.

## MATERIALS AND METHODS

### Chemistry: general

Iodomethane was from Xiya Reagent. Methyl fluorosulfonyldifluoroacetate (FSO_2_CF_2_CO_2_Me) and Iodine monochloride (ICl) was from Macklin Biochemical technology co., Ltd. Hexamethylphosphoramide (HMPA) and Copper(I) iodide were from aladding Biochemical technology co., Ltd. Anhydrous DMF was distilled under vacuum from CaH_2_. ^1^H and ^13^C NMR experiments were recorded on a Bruker AV-500 MHz spectrometer (Bruker, Karlsruhe, Germany) with tetramethylsilane (TMS) as the internal standard when DMSO-*d*_6_ was used as a solvent. Chemical shifts are expressed in *δ* (ppm) and coupling constants (*J*) in Hz. High resolution (ESI) MS spectra were recorded using an Agilent 6550 iFunnel Q-TOF LC/MS system. Pre-HPLC experiments were conducted using an Agilent 1100 HPLC system and carried on preparative Sinochrom ODS-BP (10 μm, 250×10 mm, YiLiTe, Dalian, China). HPLC analysis was used to determine the purity (> 95%) of the compounds with a YMC Pack ODS-A (5 μm, 250 × 4.6 mm, YMC Co. Ltd, Kyoto, Japan) column.

### Reagents

Quercetin was purchased from Aladdin chemistry Co. Ltd whereas TFQ was synthesized at our laboratory. Each compound was dissolved in DMSO to prepare a stock solution of 50 mM. Antibodies for the protein characterization were against total p70S6 kinase, phosphor-p70S6 kinase (Thr389), total AMPK, phosphor-AMPK (Thr172), phosphor-mTOR (S2448), total –mTOR, total 4E-BP1, phosphor-4EBP1(Thr-70) and β-actin were purchased from Cell Signaling Technology. Compound C was from Selleckchem (Houston, Texas, USA).

### Synthesis

#### Synthesis of 2-(3,4-dimethoxyphenyl)-3,5,7-trimethoxy-4H-chromen 4-one (1)

A solution of quercetin (1.51 g, 5 mmol) in mixture of anhydrous potassium carbonate and acetone (60 ml) was heated and stirred at 70°C for 30 min and then the reaction mixture was cooled to room temperature (RT). A solution of CH_3_I was added to the above mixture at room temperature. The reaction mixture was then allowed to reflux at 70°C for 24 h until TLC showed that the reaction had been completed. The reaction mixture was poured into acidic water and filtered to obtain the precipitation after wishing with H_2_O three times. The products was recrystallized from ethanol to give the pure compound (1). Yellow solid, yield 1.23 g (66.1%); ^1^H NMR (500 MHz, DMSO-*d*_6_) *δ*: 3.75 (3H, s), 3.84 (3H, s), 3.85 (3H, s), 3.86 (3H, s), 3.89 (3H, s), 6.48 (1H, d, *J* = 2.0 Hz), 6.82 (1H, d, *J* = 2.0 Hz), 7.13 (1H, d, *J* = 8.5 Hz), 7.63 (1H, d, *J* = 2.0 Hz), 7.67 (1H, dd, *J* = 8.5, 2.0 Hz); ^13^C NMR (125 MHz, DMSO-*d*_6_): 56.0, 56.1, 56.4, 56.5, 59.7, 93.4, 96.3, 108.8, 111.4, 111.9, 121.8, 122.9, 140.8, 148.8, 151.1, 152.1, 158.6, 160.7, 164.1, 172.6.

### Synthesis of 2-(3,4-dimethoxyphenyl)-8-iodo-3,5,7-trimethoxy- 4H-chromen-4- one (2)

A mixture of compound 1 (0.372 g, 1 mmol), ICl (0.485, 3 mmol) and glacial acetic acid (0.5 ml) in DMSO (10 ml) was heated with stirring at 55°C for 24 h. Then the saturated Na_2_S_2_O_3_ aqueous solution was added until the red disappeared. The resulting mixture was filtered to obtain the filtrate after washing with DMSO for three times. The filtrate was extracted with ethyl acetate until TLC showed that the no product in aqueous phase. The organic phase was washed with saturated brines, dried (anhydrous Na_2_SO_4_), and evaporated under reduced pressure to get crude product. It was further purified by prep-HPLC using ACN/H_2_O (40/60) as eluent to give compound 2. Pale yellow solid, yield 182 mg (36.5%); ^1^H NMR (500 MHz, DMSO-*d*_6_): 3.78 (3H, s), 3.85 (3H, s), 3.88 (3H, s), 3.94 (3H, s), 4.01 (3H, s), 6.66 (1H, s), 7.15 (1H, d, *J* = 8.5 Hz), 7.88 (1H, d, *J* = 2.5 Hz), 7.88 (1H, dd, *J* = 8.5, 2.5 Hz); ^13^C NMR (125 MHz, DMSO-*d*_6_): 56.1, 56.2, 56.8, 57.6, 59.6, 65.4, 93.1, 109.2, 111.4, 112.1, 122.2, 122.9, 140.6, 149.0, 151.2, 152.0, 156.2, 161.8, 163.0, 172.4; HRESIMS calcd for (C_20_H_19_IO_7_ + H)^+^ 499.0254, found 499.0252.

### Synthesis of 2-(3,4-dimethoxyphenyl)-3,5,7-trimethoxy-8- (trifluoromethyl) −4H-chromen- 4-one (3)

A solution of compound 2 (0.15 g, 0.3 mmol), CuI (37 mg), HMPA (0.3ml) in anhydrous DMF (30 ml) was heated with stirring at 75°C under strict water-free condition and nitrogen protection and then added the mixture of FSO_2_CF_2_CO_2_Me (3 ml) and anhydrous DMF (20 ml) by dripping slowly. After dripped off, the reaction mixture continue to react with stirring at 75°C overnight. The solution was cooled to RT and then added saturated NH_4_Cl aqueous solution to Quench the reaction. The reaction mixture was filtrated to remove the by-product. The filtrate was extracted with diethyl ether and wased washed 3 times with brine, dried (Na_2_SO_4_). The organic phase was evaporated to dryness in vacuo and the residue was purified by prep-HPLC using ACN/H_2_O (40/60) as eluent to give compound 3. Pale yellow solid, yield 39 mg (29.5%); ^1^HNMR (500 MHz, TMS): 3.78 (3H, s), 3.81 (3H, s),3.84 (3H, s), 4.00 (3H, s), 4.04 (3H, s), 6.71 (1H, s), 7.16 (1H, d, *J* = 8.0 Hz), 7.60 (1H, d, *J* = 2.0 Hz), 7.69 (1H, dd, *J* = 8.0, 2.0 Hz); ^13^C NMR (125 MHz): 55.7, 56.0, 57.1, 57.6, 59.7, 93.2, 108.6, 110.8, 112.2, 122.0, 122.4, 125.3, 130.0, 140.8, 148.8, 151.3, 152.1, 155.7, 162.7, 164.0, 172.0; ^19^F NMR (471 MHz): −52.5; HRESIMS calcd for (C_21_H_19_F_3_O_7_ + H)^+^ 441.1161, found 441.1162.

### Cell lines and culture conditions

Murine and human bladder cancer cell provided by Dr. P Guo (Institute of Urology, Xi'an Jiaotong University, Xi'an, Shanxi, China) was cultured in DMEM supplemented (Hyclone, Logan, UT, USA) with 10% of FBS (Hyclone, Logan, UT, USA) and 1% of penicillin-streptomycin at 37°C, in humidified air containing 5% of CO_2_.

### Cell viability assay

Cell viability was assessed using a tetrazoliumbased assay using microplate reader (Biotek, SYNERGY HTX, Vermont, USA). IC50 values were determined through the dose-response curves.

Cells were seeded at 6 × 10^3^ per well in 96-well culture plates and incubated in medium containing 10% FBS. Different seeding densities were optimized at the beginning of the experiments. After 24 h, cells were treated with different concentrations of Que or TFQ (0, 5 μM, 10 μM, 20 μM, 40 μM, 80 μM, 160 μM) for 48 hours in incubator. 50 μl of MTT tetrazolium salt (Sigma) dissolved in Hank's balanced solution at a concentration of 2 mg/ml was added to each well with indicated treatment and incubated in CO_2_ incubator for 5 h. Finally, the medium was aspirated from each well and 150 μl of DMSO (Sigma) was added to dissolve formazan crystals and the absorbance of each well was obtained using a previously stated plate reader.

### Clonogenic assay

Cologenic survival was defined as the ability of the cells to form colonies. Images were taken and analyzed by microscopy (Leica, DFC450C, Wetzlar, Germany) and microplate reader (Biotek, SYNERGY HTX, Vermont, USA).

8 × 10^3^ cells were seeded into 24-well dishes in 0.5 ml of medium. Twenty-four hours later, cells were treated with different concentrations of Que or TFQ (0 μM, 2.5 μM, 5 μM, 10 μM, 20 μM), and then maintained for 6 days in a CO_2_ incubator Finally, the cells were fixed with 10% formaldehyde solution and then stained with 0.1% crystal violet, each experiment was performed in triplicate and colony numbers calculated through spectral scanning to determine the maximum absorption wavelength at 550 nm and measured its absorbance through area scanning. Statistical analysis was prepared with the GraphPad Prism 6.0. The Student's t testwas used to compare colony numbers and determine the significance of these differences.

### Measurement of cell migration

A total of 5 × 10^3^ cells were seeded onto a six-well plate and allowed to reach full confluence. The monolayer was wounded using a cocktail stick. Cells were incubated with serum-free DMEM medium, for the time period as stated. Digital images were taken at times 0, 24 h and 48 h. The mean area was calculated using Image J software. The experiments were repeated three times.

### Assessment of apoptosis

Apoptosis was detected by flow cytometry via the examination of altered plasma membrane phospholipid packing by lipophilic dye Annexin V. Briefly, treated cells were harvested by trypsin, washed twice with PBS, and then resuspended in binding buffer at a concentration of 1 × 10^6^ cells/mL according to the manufacturer's instruction. Thereafter, 5 μL of Annexin V-FITC and 10 μL of propidium iodide were added into 100 μL of cell suspension and incubated for 30 minutes at room temperature in the dark. After adding 400 μL of binding buffer, labeled cells were counted by flow cytometry within 30 minutes. All early apoptotic cells (Annexin V–positive, propidium iodide–negative), necrotic/late apoptotic cells (double positive), as well as living cells (double negative) were detected by FACSC alibur flow cytometer and subsequently analyzed by Cell Quest software (Becton Dickinson). Argon laser excitation wavelength was 488 nm, whereas emission data were acquired at wavelength 530 nm (FL-1 channel) for fluorescein isothiocyanate (FITC) and 670 nm (FL-3 c3 channel) for propidium iodide.

### Protein characterization

Western blot assessment was performed using regular procedure. Primary antibody was added in BSA and allowed to incubate overnight at 4°C, washed with TBS/0.05% Tween-20 for 5 times (10 min per time) before the secondary antibody was added and then incubated for an additional hour at room temperature. The membrane was again washed 3 times before adding Pierce Super Signal chemiluminescent substrate (Rockford, IL, USA) and then immediately imaged on Chemi Doc (Bio-Rad, Hercules, CA, USA). The films were scanned using EPSON PERFECTION V500 PHOTO and quantified by Image J (NIH, Bethesda, MD, USA).

### Statistical analysis

Statistical analysis was performed using SPSS 16.0 software. Student's *t* test was used to analyze the significance of two group results. Graphs were prepared using GraphPad Prism 6.0. All data are presented as mean ± SD. Statistical analyses were carried out using one-way ANOVA analysis and statistical significance was assumed at a value of *p < 0.05, **P < 0.01.

## SUPPLEMENTARY MATERIALS FIGURES


