# Supplementary material for: A novel synthetic derivative of quercetin, 8-trifluoromethyl-3,5,7,3′,4′-O-pentamethyl-quercetin, inhibits bladder cancer growth by targeting the AMPK/mTOR signaling pathway
Source: Oncotarget. 2017 May 11;8(42):71657–71. doi: 10.18632/oncotarget.17799 (PMC5641079; doi:10.18632/oncotarget.17799)
Supplement: Supplementary file 1 [file oncotarget-08-71657-s001.pdf]

A novel synthetic derivative of quercetin, 8-trifluoromethyl-3,5,7,3′,4′-*O*-pentamethyl-quercetin, inhibits bladder cancer growth by targeting the AMPK/mTOR signaling pathway

Supplementary Materials

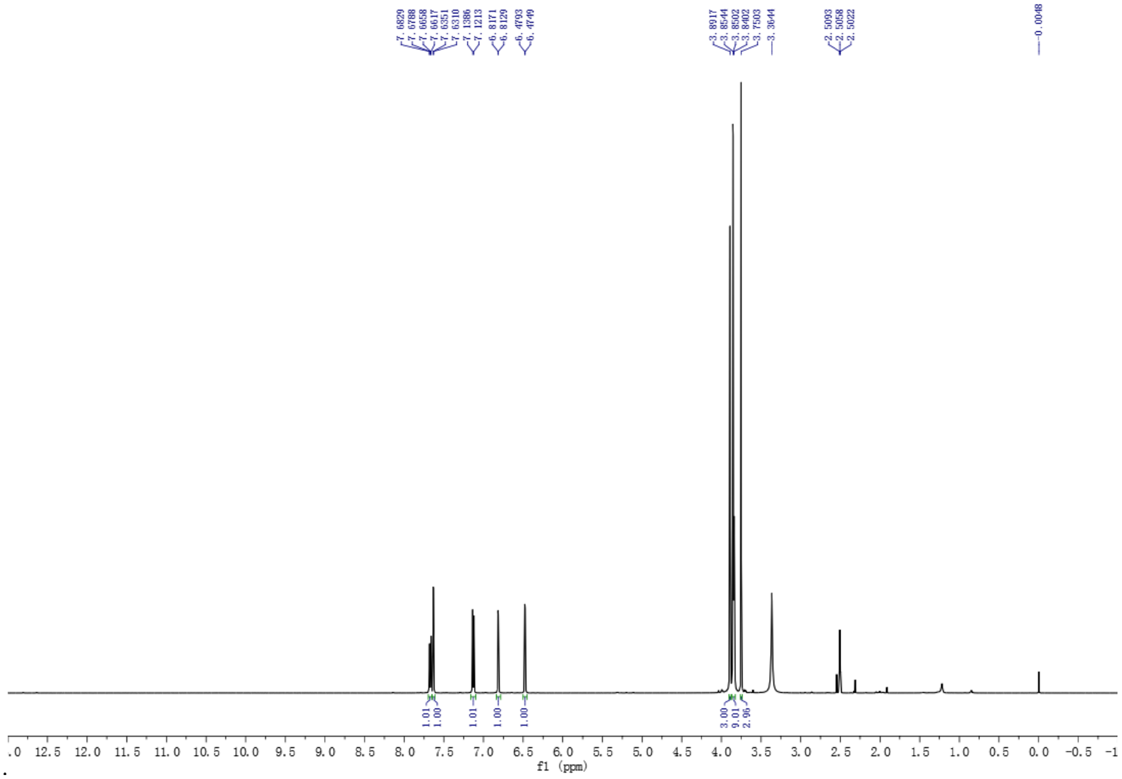

Supplementary Figure 1: ^{1}H NMR spectrum of compound 1.

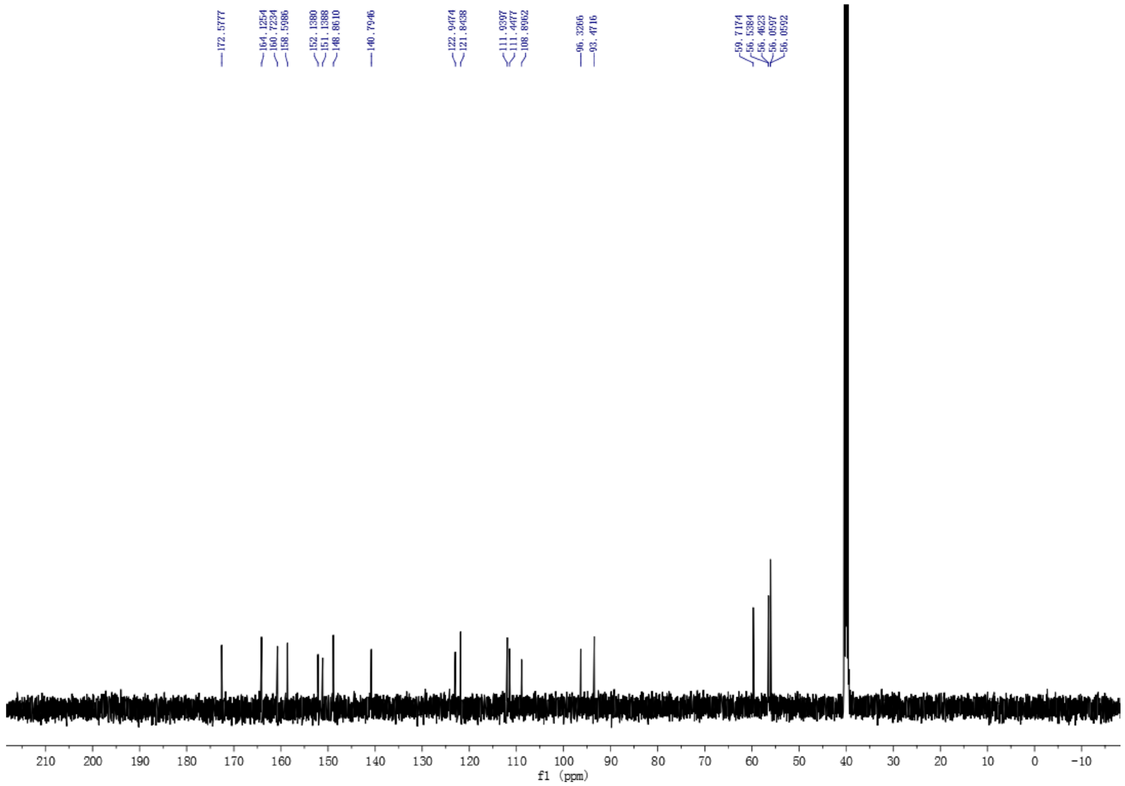

Supplementary Figure 2: ^{13}C NMR spectrum of compound 1.

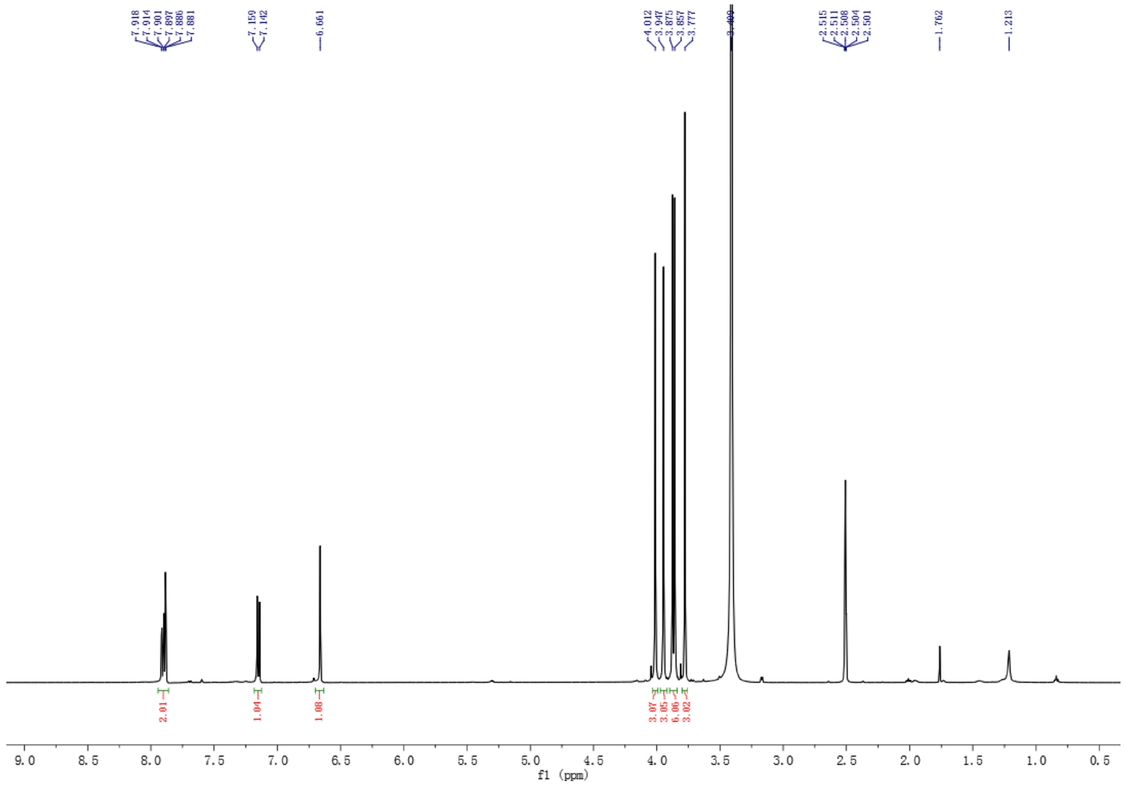

Supplementary Figure 3: ^{1}H NMR spectrum of compound 2.

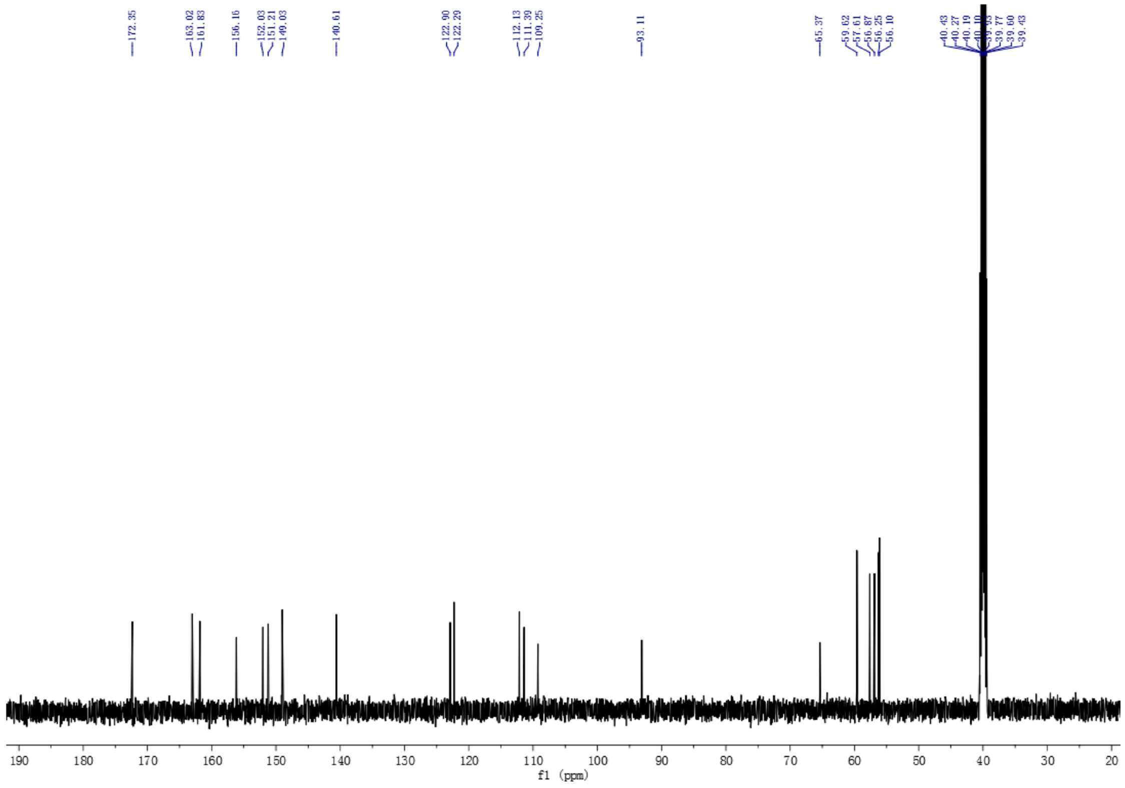

Supplementary Figure 4: ^{13}C NMR spectrum of compound 2.

Qualitative Analysis Report

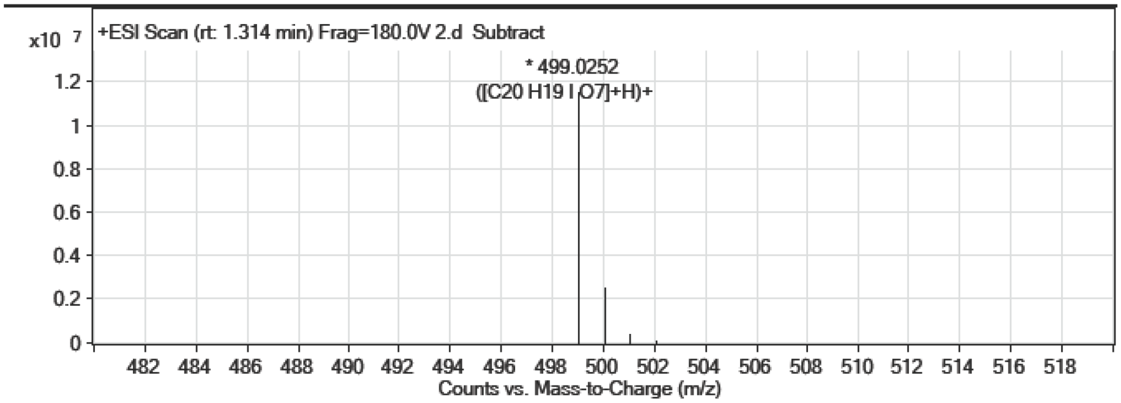

Peak List

m/z	z	Abund	Formula	Ion
499.0252	1	11501117	C20 H19 I O7	(M+H)+
500.028	1	2475195.5	C20 H19 I O7	(M+H)+
1019.0241	1	3039237.75		
1020.0277	1	1277137.5		

Formula Calculator Element Limits

Element	Min	Max
C	3	60
H	0	120
O	0	30
N	0	0
S	0	5
Cl	0	3
F	0	5
I	0	1

Formula Calculator Results

Formula	Best	Mass	Tgt Mass	Diff (ppm)	Ion Species	Score
C20 H19 I O7	True	498.0178	498.0175	-0.46	C20 H20 I O7	99.49
C17 H20 F I O8	False	498.0178	498.0187	1.82	C17 H21 F I O8	96.5
C18 H21 F2 I O4 S	False	498.018	498.0173	-1.31	C18 H22 F2 I O4 S	92.57
C15 H19 F4 I O6	False	498.0178	498.0162	-3.09	C15 H20 F4 I O6	90.14
C15 H22 F3 I O5 S	False	498.018	498.0185	0.95	C15 H23 F3 I O5 S	89.61
C25 H7 F5 O6	False	498.0178	498.0163	-3	C25 H8 F5 O6	89.58
C27 H8 F2 O8	False	498.0178	498.0187	1.91	C27 H9 F2 O8	88.18
C25 H10 F4 O5 S	False	498.0179	498.0185	1.14	C25 H11 F4 O5 S	87.72
C21 H20 F I O3 S	False	498.018	498.0162	-3.58	C21 H21 F I O3 S	87.29
C12 H20 F5 I O7	False	498.0178	498.0174	-0.81	C12 H21 F5 I O7	87.16

--- End Of Report ---

Supplementary Figure 5: HRESIMS of compound 2.

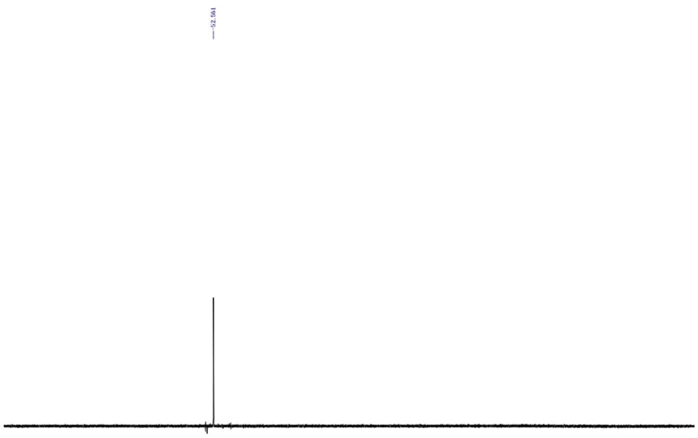

Supplementary Figure 6: ^{19}F NMR spectrum of compound 3.

Qualitative Analysis Report

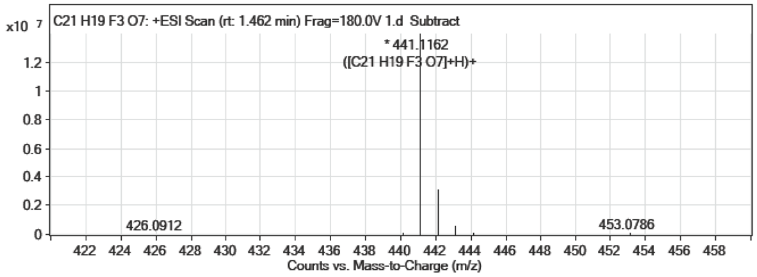

Peak List

m/z	z	Abund	Formula	Ion
441.1162	1	13966631	C21 H19 F3 O7	(M+H)+
442.1187	1	3119553.25	C21 H19 F3 O7	(M+H)+
443.1216	1	515871	C21 H19 F3 O7	(M+H)+
463.0973	1	279438.53		
499.0243	1	295311.72		
903.2058	1	2633977.75		
904.2094	1	1178994.75		
905.2121	1	312753.16		
915.1692	1	155185.2		
916.1727	1	70945.27		

Figure S4. ^{13}C NMR spectrum of compound **2**.

Formula Calculator Element Limits

Element	Min	Max
C	3	60
H	0	120
O	0	30
N	0	0
S	0	5
Cl	0	3
F	0	5

Formula Calculator Results

Formula	Best	Mass	Tgt Mass	Diff (ppm)	Ion Species	Score
C15 H11 F N13 O3	False	440.1091	440.1092	0.22	C15 H12 F N13 O3	99.14
C17 H14 F4 N7 O3	False	440.1089	440.1094	1.11	C17 H15 F4 N7 O3	99
C16 H17 F N6 O8	False	440.1089	440.1092	0.6	C16 H18 F N6 O8	98.63
C21 H19 F3 O7	True	440.1088	440.1083	-1.1	C21 H20 F3 O7	98.57
C21 H18 N3 O8	False	440.1088	440.1094	1.26	C21 H19 N3 O8	98.15
C19 H16 N6 O7	False	440.1089	440.108	-1.94	C19 H17 N6 O7	98.06
C20 H13 F3 N7 O2	False	440.1089	440.1083	-1.43	C20 H14 F3 N7 O2	97.95
C20 H12 N10 O3	False	440.109	440.1094	0.94	C20 H13 N10 O3	97.54
C18 H20 F4 O8	False	440.1088	440.1094	1.49	C18 H21 F4 O8	97.11
C15 H12 F4 N10 O2	False	440.109	440.1081	-2.15	C15 H13 F4 N10 O2	96.45

Fragmentor Voltage	Collision Energy	Ionization Mode
180	0	ESI

Agilent Technologies

Supplementary Figure 7: HRESIMS of compound 3.

MB49

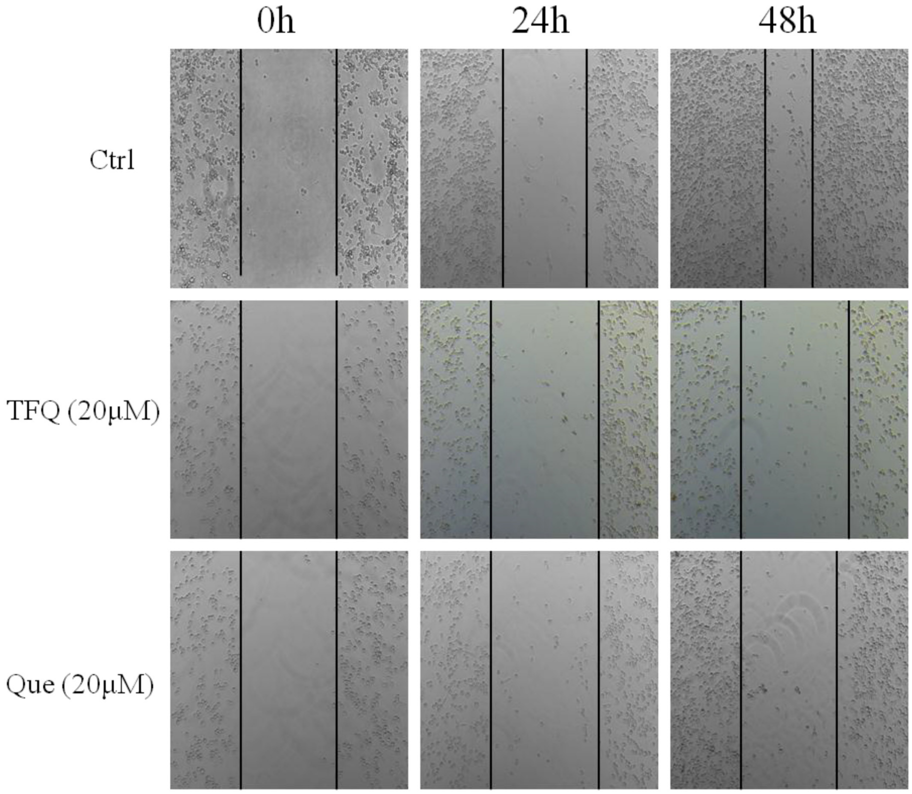

0h
24h
48h
Ctrl
TFQ (20μM)
Que (20μM)

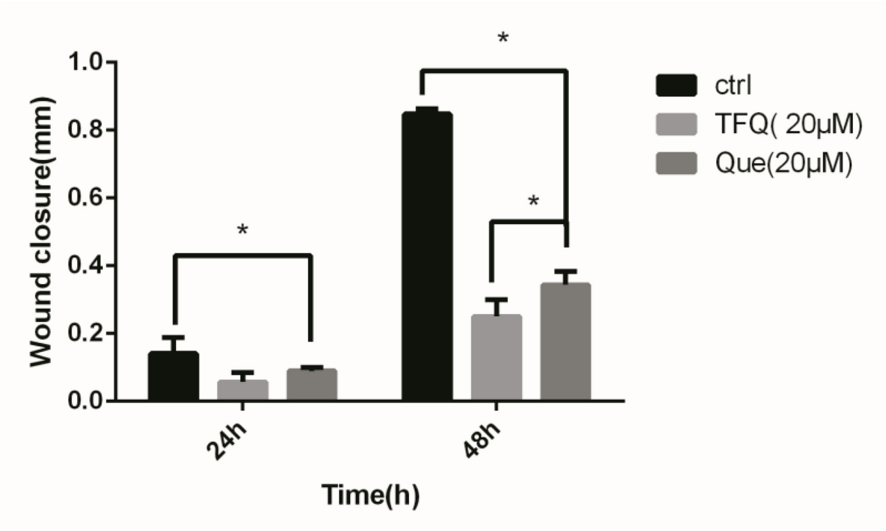

1.0
0.8
0.6
0.4
0.2
0.0
Wound closure(mm)
*
*
*
24h
48h
Time(h)
ctrl
TFQ(20μM)
Que(20μM)

T24

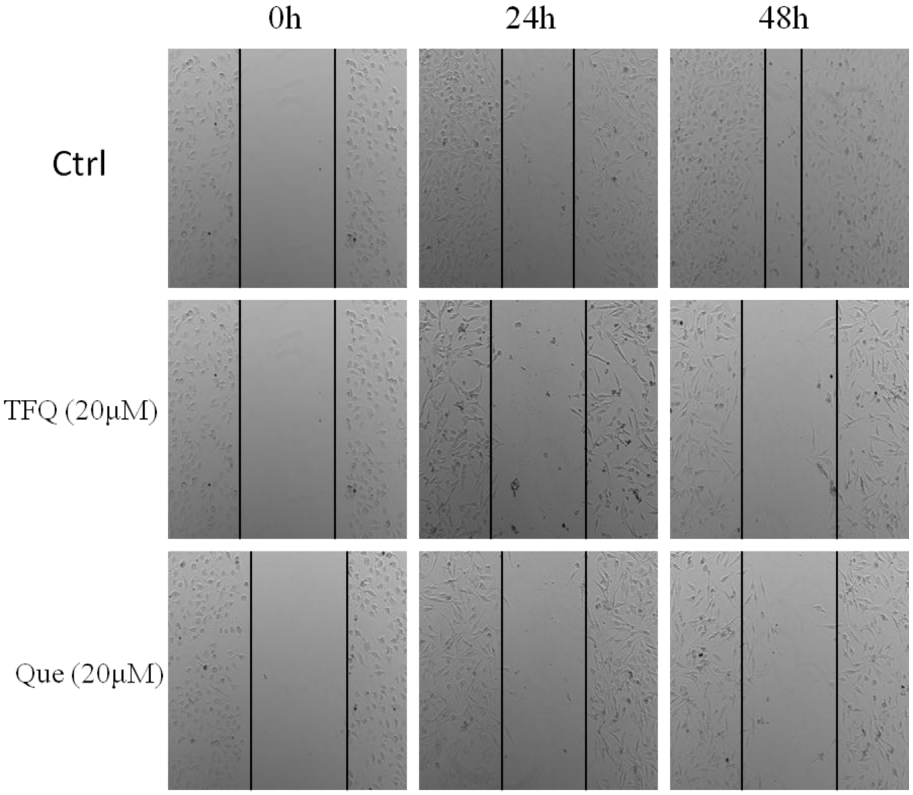
0h
24h
48h
Ctrl
TFQ (20μM)
Que (20μM)

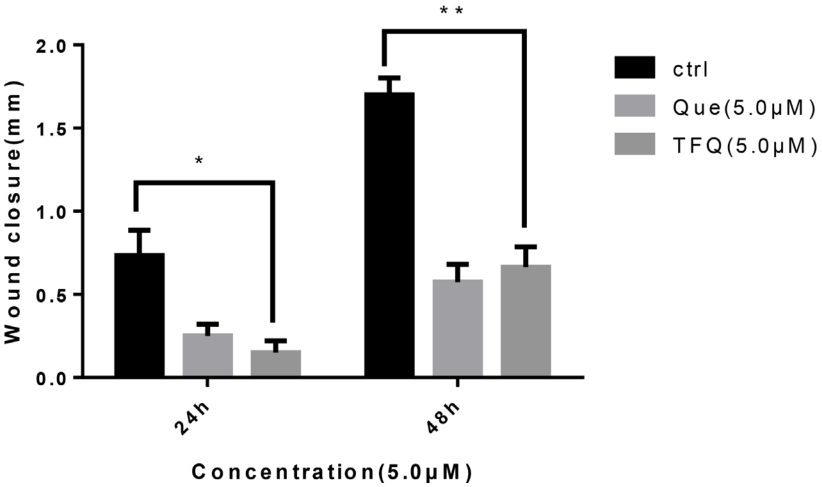
Wound closure(mm)
2.0
1.5
1.0
0.5
0.0
*
**
24h
48h
Concentration(5.0μM)
ctrl
Que(5.0μM)
TFQ(5.0μM)

UMUC3

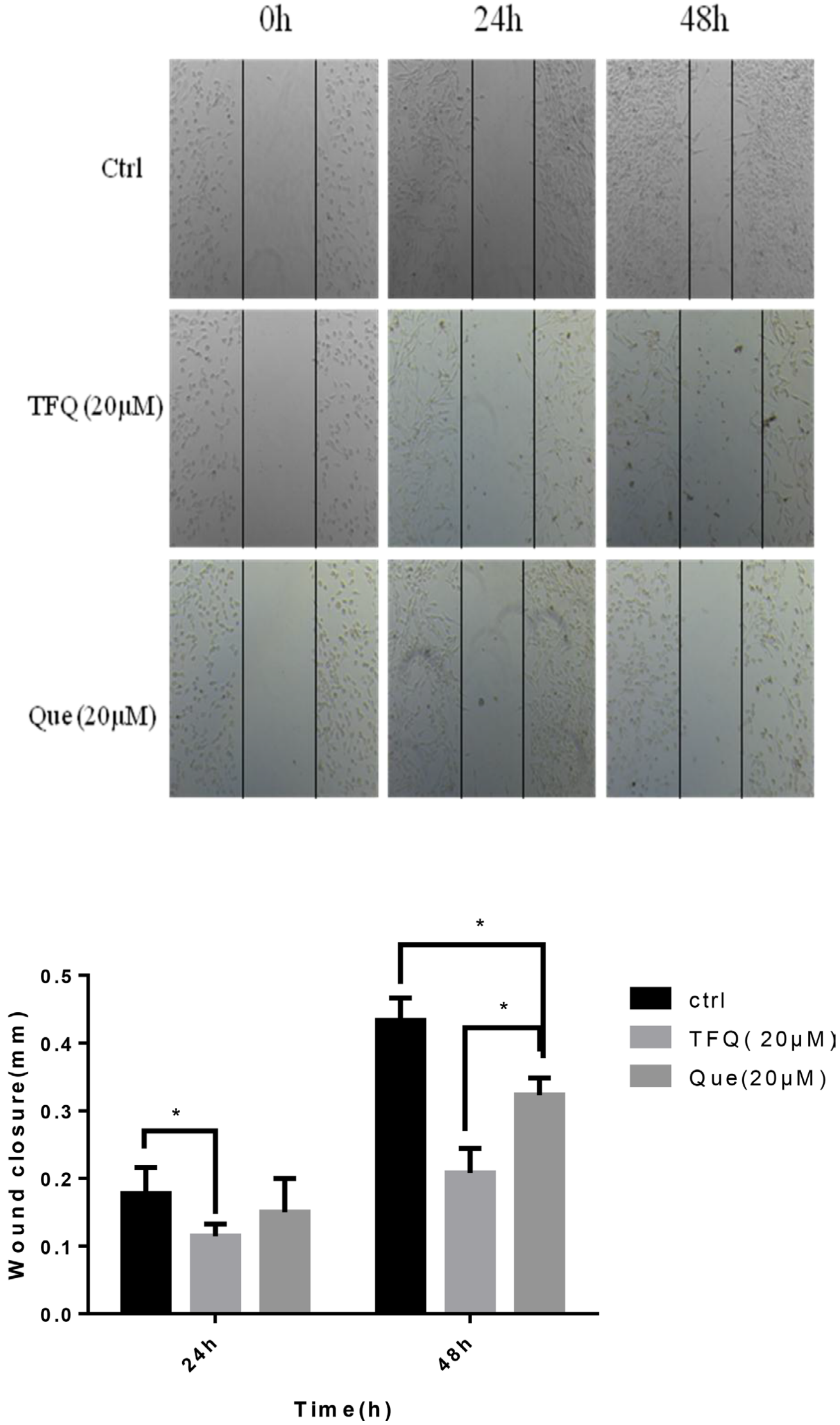

Supplementary Figure 8: TFQ or Que treatment showed impaired migration in wound healing assays. (**A–C**) Images showed the gap of the scratched region of different cells with the treatment of 20 μM TFQ or Que; A. Wound healing assays in MB49. Above: The images were taken through an inverted microscope with ×10 magnification. Below: The mean area was calculated using Image J software. Results are presented as the median of 5 independent experiments (**P* < 0.05 vs control and TFQ vs Que). (**B–C**) Wound healing assays was conducted with the treatment at 20 μM TFQ or Que in T24 and UMUC3, respectively. The images and their mean area were obtained through the same method as described in MB49.
